# The impact of a multidomain lifestyle and empowerment program for mild cognitive impairment on care partner wellbeing

**DOI:** 10.1002/alz70858_103396

**Published:** 2025-12-25

**Authors:** Amy D. Rodriguez, Kayci L. Vickers, Felicia C. Goldstein, Liselotte De Wit, Jessica L. Saurman, Emily L. Giannotto, Jacquelyn Thelin, Allan I. Levey

**Affiliations:** ^1^ Emory University School of Medicine, Atlanta, GA, USA

## Abstract

**Background:**

Individuals with mild cognitive impairment (MCI) experience cognitive and functional declines that impact their ability to complete everyday activities. Spouses or family members often take on a care giving role, which can lead to depression, stress, and caregiver burden. Expanding access to interventions aimed at reducing lifestyle risk factors and improving quality of life for individuals and families facing cognitive decline is a national priority. Yet, multidomain intervention programs are still not widely available. The Cognitive Empowerment Program (CEP) was developed to address this critical gap. The objective of this study was to evaluate the impact of CEP on care partner wellbeing.

**Method:**

Participants were 94 care partners (67 females; mean age = 69.07 years, SD = 10.81) of individuals with a diagnosis of MCI from Emory's Cognitive Neurology Clinic. Care partners were given the option to participate with their care recipient in a 12‐month program comprising physical, cognitive, and psychosocial interventions, as well as care partner education and support groups. Questionnaires were administered at baseline and after program completion to assess depression (Center for Epidemiological Studies‐ Depression; CES‐D), stress (Perceived Stress Scale; PSS), caregiver burden (Zarit Burden Interview– 12‐item; ZBI‐12), and empowerment (Care Partner Empowerment Scale). Data were analyzed using descriptive statistics and paired samples t‐tests.

**Result:**

Upon program entry, care partners reported low stress (PSS mean = 11.25, SD = 6.14), mild burden (ZBI‐12 mean = 12.63, SD = 7.96), and non‐clinically significant symptoms of depression (CES‐D mean = 11.75, SD = 7.59). At program exit, there was no significant change in depressive symptoms (t(84) = 0.03, *p* = .97) or stress (t(84) = ‐0.99, *p* = .33). Care partners reported a significant increase in caregiver burden, but it remained in the mild range (t(83) = ‐2.27, *p* = .03) and was accompanied by a significant increase in empowerment (t(76) = ‐2.89, *p* < .01).

**Conclusion:**

Care partners enrolled in CEP demonstrated an improvement in their feelings of empowerment despite a slight worsening of caregiving burden over one year. These results suggest that a multidomain lifestyle and empowerment program can positively impact care partner wellbeing. Further research is needed to identify both patient and care partner characteristics that affect wellbeing outcomes.

